# SEDATION IN COLONOSCOPY BY USING THREE DIFFERENT PROPOFOL INFUSION METHODS AND ANALYSIS OF PLASMA CONCENTRATION LEVELS: A PROSPECTIVE COMPARATIVE STUDY

**DOI:** 10.1590/0102-6720201600040012

**Published:** 2016

**Authors:** Paulo Henrique Boaventura de CARVALHO, José Pinhata OTOCH, Mohamad Ali KHAN, Paulo SAKAI, Hugo Gonçalo GUEDES, Everson Luiz de Almeida ARTIFON

**Affiliations:** 1 Anesthesiology Division,; 2 Department of Surgery,; 3 Division of Gastrointestinal Endoscopy, School of Medicine, University of São Paulo, São Paulo, SP, Brazil; 4 Division of Gastroenterology, University of Tennessee Health Science Center, Memphis, TN, 38163, USA.

**Keywords:** Propofol, Anesthesia, Deep sedation, Colonoscopy, Capnography

## Abstract

**Background:**

The propofolemia becomes directly linked to the clinical effects of this anesthetic and is the focus for studies comparing propofol clinical use, in different administration methods routinely used in endoscopy units where sedation is widely administered to patients.

**Aim:**

To evaluate the effects of three different regimens of intravenous propofol infusion in colonoscopies.

**Methods:**

A total of 50 patients that underwent colonoscopies were consecutively assigned to three groups: 1) intermittent bolus infusion; 2) continuous manually controlled infusion; 3) continuous automatic infusion. Patients were monitored with Bispectral Index^TM^ (BIS) and propofol serum levels were collected at three different timepoints. The development of an original dilution of propofol and an inventive capnography catheter were necessary.

**Results:**

Regarding clinical outcomes, statistical differences in agitation (higher in group 1, p=0.001) and initial blood pressure (p=0.008) were found. As for propofol serum levels, findings were similar in consumption per minute (p=0.748) and over time (p=0.830). In terms of cost analysis, group 1 cost was R$7.00 (approximately US$2,25); group2, R$17.50 (approximately US$5,64); and group 3, R$112.70 (approximately US$36,35, p<0.001). Capnography was able to predict 100% of the oxygen saturation drop (below 90%).

**Conclusion:**

The use of propofol bolus administration for colonoscopies, through continuous manually controlled infusion or automatic infusion are similar regarding propofolemia and the clinical outcomes evaluated. The use of an innovative capnography catheter is liable and low-cost solution for the early detection of airway obstruction.

## RESUMO

**Racional:**

A propofolemia está diretamente relacionada com os efeitos clínicos desse anestésico e é foco de diversos estudos comparando os usos clínicos do propofol e os diferentes métodos de administração, como realizado amplamente nos centros de endoscopia.

**Objetivo:**

Avaliar os efeitos de três diferentes regimes de infusão de propofol intravenoso em colonoscopias.

**Métodos:**

Ao todo 50 pacientes que foram submetidos à colonoscopia foram consecutivamente divididos em três grupos: 1) infusão em bolus intermitente; 2) perfusão contínua controlada manualmente; 3) infusão automática contínua. Os pacientes foram monitorados com Bispectral Index^TM^ (BIS) e os níveis séricos de propofol foram coletados em três momentos diferentes. Foi necessário a preparação de uma diluição específica de propofol e o desenvolvimento de um cateter de capnografia original manufaturado para a realização do estudo.

**Resultados:**

Em relação aos desfechos clínicos, houve diferença estatística na agitação (maior no grupo 1, p=0,001) e pressão arterial inicial (p=0,008). Com relação aos níveis séricos de propofol, os resultados foram semelhantes no consumo por minuto (p=0,748) e ao longo do tempo (p=0,830). Em termos de análise de custo, no grupo 1 o custo foi de R$ 7,00 (aproximadamente US$ 2,25); grupo 2, R$ 17,50 (aproximadamente US$ 5,64); e grupo 3, R$ 112,70 (cerca de US$ 36,35, p<0,001). A capnografia foi capaz de diagnosticar 100% das dessaturações de oxigênio (abaixo de 90%).

**Conclusão:**

O uso de propofol em bolus para colonoscopias, por meio de infusão contínua controlada manualmente ou infusão automática são semelhantes quanto à propofolemia e os resultados clínicos avaliados. Além disso, o uso de um cateter de capnografia inovador é solução de baixo custo para a detecção precoce da obstrução da via aérea.

## INTRODUCTION

Propofol pharmacokinetic model is related to cardiac output and its fast distribution to tissues. This effect is closely related to its plasma concentration that rapidly decreases after the drug is administered^5^. Due to this peculiarity, propofolemia becomes directly linked to the clinical effects of this anesthetic and the focus of studies comparing propofol clinical use, in different administration methods routinely used in endoscopy units where sedation is widely administered to patients^10^.

Because of the particularities of every patient, different sedation levels are required for every procedure examination at specific time points, so that the titrated administration of propofol is a topic of interest^7^.

Accordingly, three different sedation regimens in colonoscopy are compared in the present study, evaluating clinical and laboratory parameters to determine differences in propofol administration for colonoscopy sedation.

## METHODS

### Patients and method

Patients scheduled to undergo colonoscopy routinely in the Endoscopy Unit of Hospital das Clínicas, University of São Paulo Medical School in São Paulo and in the Endoscopy Unit at Hospital Ana Costa in Santos, both in São Paulo state, Brazil, from April 2013 to December 2014, were selected for this study. This project was granted approval by the Ethics Committees of both units, and was registered in the São Paulo University Ethics Committees by number 1086/06, and was registered in the Hospital Ana Costa Ethics Committees by number 021/12.

Selection and assignment of patients were consecutive subjected to the colonoscopy schedule of the participating institutions. A different group was performed for every daily routine and, since the number of anesthesia procedures per day differed, anesthesia procedures were randomly performed in one or more groups in order to match the final sample number. Patients with clinical condition classified as ASA class IV or higher were excluded. They were monitored by continuous electrocardiogram, pulse oximetry, aspiration capnography, noninvasive blood pressure devices and bispectral sensors (BIS). All patients received supplementary 3 l/min of nasal catheter oxygenation. 

**FIGURE 1:**
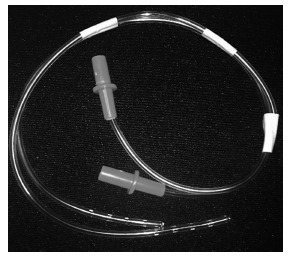
Innovative crafted capnography/oxygenation catheter

Capnography was performed using aspiration type monitors. Additionally, an innovative gauging system was crafted by using a number-8 nasal catheter immediately prepared prior to every examination and attached to an identical catheter used to administer oxygen (O_2_) at a 4 cm interval between its tips, so that the O_2_ tip was proximal (Figure 1).

Regarding BIS monitoring, XP 2000 Aspect Medical Rev 3.01 (Aspect Medical Systems, Newton, MA) were used. 

Blood samples were preserved under refrigeration and analyzed by a high performance liquid chromatographer that produced serum dosage for propofol analysis.

The first propofol serum concentration was collected 5 min after induction, and the second when the cecum was reached. The third was collected at examination completion, and finally, 5 min from the last propofol dose.

Three schemes protocols for administering sedation were used, as follows:

Group 1: Firstly, a dose of fentanyl at 1 µg/kg was administered. One minute after fentanyl administration, an initial propofol dose at 1 mg/kg was injected over 1 min. Subsequently, the colonoscope was introduced and sedation with 30 mg bolus according to the clinical need of every patient was complemented, as agitation or movement, or the need for deepening anesthetic level to perform surgical procedures during colonoscopy, disregarding BIS values from every patient. Time points in which intermediary doses were administered were recorded along with the events that lead to a new dose.

Group 2: An innovative solution was prepared for the examination and was comprised of 0.2% propofol diluted in 5% glucose solution. Solutions were prepared immediately before every examination began and were weighted in order to evaluate the dosage of propofol used. Initially a fentanyl dose at 1 µg/kg was administered, similarly to group 1. One minute after fentanyl administration, an initial propofol dose at 1 mg/kg was injected over 1 min (for dilution purposes, every patient weight in ml was halved). After this initial dose, a continuous infusion of propofol at approximately 100 µg/kg/min was started which, for this specific solution, was equivalent to approximately 1 drip kg/min. Solution dripping varied across clinical parameters of patients reaching examination needs. In case of reaction of a patient to the drug became of note or an immediate deepening was required, an intermediary bolus dose of 30 mg (15 ml of solution) was administered from the infusion vial itself.

Group 3: This group was comprised of patients that underwent target-controlled infusion (TCI) (Diprifusor^(r)^ [Fresenius, Brezins, France]) and the drug was standardized for all patients (Diprivan PFS^(r)^ [Corden Pharma, Monza-Brianza, Italy]) - pre-prepared syringe with 50 ml propofol at 1%).

A dose of 1 µg/kg of fentanyl was injected at first, similarly to groups 1 and 2. Subsequently, propofol intravenous infusion was initiated, previously programmed according to the weight, gender and age of patients, with a pre-programmed induction of 1 min up to the target dose of 4 µg/ml, and after this initial dose was injected, the infusion pump was reprogrammed at 2 µg/ml.

Similarly to the other two groups, this dosage was adjusted according to the clinical parameters for procedures. These dose changes were reported at the moment they were performed, as well as the criteria adopted for this change.

In order to evaluate the cost of procedures, spread sheets for every group were individually generated for every item related to propofol use because only this anesthetic was used differently in all groups.

### Cost analysis

Pricing was obtained by using both the Brazilian publication (Brasíndice)^3^ in accordance with the National Health Surveillance Agency (Agência Nacional de Vigilância Sanitária - ANVISA)^11^, as well as from the direct contact with medical material suppliers yielding the following values for the three groups.

#### Group 1 

For every 200 mg of propofol used during procedures in this group, the estimated cost for listed materials and drugs were as follows: A 200 mg vial-ampoule of propofol at 1% cost the average price of R$ 4.00 (approximately US$ 1,29). A 20 ml syringe cost the average price of R$ 0.31 (approximately US$ 0,01). A 30x12 needle for drug aspiration cost the average price of R$ 0.58 (approximately US$ 0,19).

#### Group 2 

For every 600 mg of propofol used during procedures in this group, the estimated cost for listed materials and drugs were as follows: three 200 mg vial-ampoules of propofol at 1% totaling 600 mg for the average cost of R$12.00 (approximately US$ 4.06). A 20 ml syringe cost the average price of R$ 0.31 (approximately US$ 0.01). A 30x12 needle for drug aspiration cost the average price of R$ 0.58 (approximately US$ 0.19). A 250 ml bottle of glucose solution at 5% cost the average price of R$ 2.35 (approximately US$ 0.75). An infusion set for glucose solution to administer propofol cost the average price of R$ 2.55 (approximately US$ 0.82).

#### Group 3 

For every 500 mg of propofol used during procedures of this group, the estimated cost for listed materials and drugs were: a 500 mg syringe of Diprivan PFS^(r)^ at 1% used in the TCI Diprifusor^(r)^ pump cost the average price of R$104.60 (approximately US$ 33.74). A plastic extension for connecting Diprivan PFS to a three-way tap cost the average price of R$ 2.92 (approximately US$ 0.94). 

### Statistical analysis

With the assumption that differences in propofol plasma concentration would be found at the end of procedures of at least a standard deviation between the best and the worst group tested, the minimum sample required to conduct this study with 95% confidence and 80% power was determined to be 17 patients for each group, based on the comparison of means for the three groups using analysis of variance (ANOVA)^12^. Quantitative features were described according to groups using mean, standard deviation (SD), median, minimum, and maximum and they were compared across groups by using ANOVA test. Gender and ASA physical status were described across groups using absolute and relative frequencies. Additionally, an association of gender with groups using likelihood ratio was verified^6^ and ASA class was compared across groups by performing Kruskal-Wallis test^12^. Measurement of propofol serum levels was described according to infusion type and evaluation time points using mean and standard deviation, as evidenced by graphic mean profiles for infusion types and evaluation time points using ANOVA with repeated measures and two factors followed by Bonferroni multiple comparisons^12^ to verify in which interval of time points propofol serum level differences occurred. All tests were conducted with significance level at 5%. General clinical characteristics of all 50 patients participating in this study were found to be statistically similar to personal characteristics evaluated and through ASA classification (p>0.05).

## RESULTS

Patients were evaluated regarding their demographic characteristics (Table 1) and propofol serum concentration was evaluated 5 min after infusion, during procedure (intubation of cecum) and at the completion of procedure. Furthermore, propofol cost, the lowest BIS value reached after induction and time to reach the lowest value, examination duration, number of agitations per hour, oxygen saturation drop below 90% within an interval no greater than 5 min, airway obstruction occurrences perceived by changes in the capnographic curves of patients evaluated, and the need for using scopolamine to ease examination, requested by the endoscopist, were evaluated as well.

**TABLE 1:** Demographic characteristics evaluated in patients according to infusion types

Variable	Group1 (n=16)	Group 2 (n=17)	Group 3 (n=17)	Total (n=50)	p
Gender, n (%)					0.976
Female	9 (56.2)	9 (52.9)	9 (52.9)	27 (54)	
Male	7 (43,8)	8 (47.1)	8 (47.1)	23 (46)	
ASA, n (%)					0.945#
I	5 (31.2)	6 (35.3)	5 (29.4)	16 (32)	
II	10 (62.5)	10 (5.,8)	11 (64.7)	31 (62)	
III	1 (6.2)	1 (5.9)	1 (5.9)	3 (6)	
Age (years)					0.896**
mean (SD)	51.9 (11.5)	54.2 (16)	53.2 (14,4)	53.1 (13.9)	
Weight (Kg)					0.340**
mean (SD)	70.7 (12.9)	71.4 (14.8)	77,5 (15,7)	73,2 (14,6)	
Height (m)					0.947**
mean (SD)	1.65 (0.11)	1.64 (0.11)	1,65 (0,1)	1.65 (0.1)	
BMI (Kg/m2)					0.406**
mean (SD)	26.1 (4.3)	26.5 (5.1)	28.2 (5.1)	26.9 (4.8)	

Chi-square test; #Kruskal-Wallis test; **ANOVA; ASA=American Society of Anesthesiology; SD=standard deviation; min=minimum; max=maximum.

### Analysis of propofol serum levels

No differences in propofol serum levels across infusion regimens used in this study were found (Table 2) and reduction in propofol serum levels along the procedure for all infusion types was demonstrated.

**TABLE 2:** Propofol serum concentration data according to infusion types along evaluation time points

Time point	Group 1			Group 2				Group 3	
	Mean	SD	n	Mean	SD	n	Mean	SD	n
5 min after Infusion	3.23	0.88	16	3.12	0.89	17	3.24	0.87	17
During procedure	2.15	0.68	16	2.12	0.73	17	2.14	0.76	17
End of procedure	1.07	0.41	16	1.20	0.53	17	1.21	0.48	17

SD=standard deviation

Mean values for infusion types were found to be statistically similar along evaluation time points (p=0.830) and no mean statistical difference was verified among infusion regimens (p=0.964). However, mean statistical difference in propofol plasma concentration was found between evaluation time points regardless of the infusion type used (p<0.001).

Mean propofol concentration levels were verified to be statistically reduced at every evaluation time point in both types of infusion (p<0.001, Table 3).

**TABLE 3:**
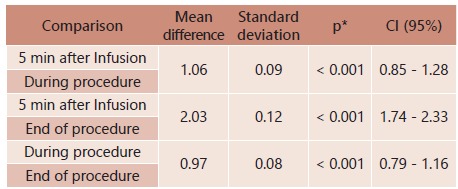
Multiple comparison results for propofol serum concentration along time points

*.: Bonferroni multiple comparisons

Analysis of clinical parameters and drug consumption during examination

No significant difference was found in mean propofol consumption across groups, between lowest BIS mean value reached, examination mean duration, use of scopolamine, airway obstruction episodes, and episodes of oxygen saturation below 90%, although no episode occurred in group 1(Table 4).

**TABLE 4:**
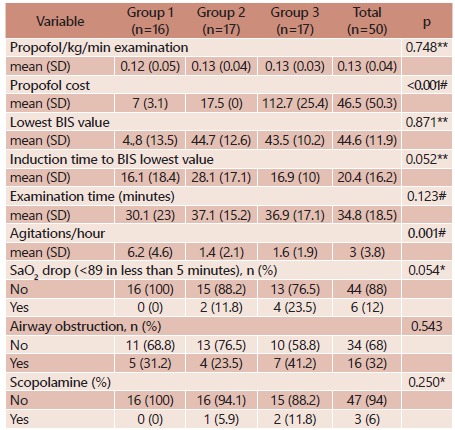
Parameter evaluated during procedure across groups and results from comparative tests

Chi-square test; * Likelihood ration test; # Kruskal-Wallis test; ** ANOVA; SD=standard deviation; min=minimum; max=maximum

Agitations per hour were observed to be statistically higher in group 1 than in remaining groups (p<0.005). Nonetheless, this variable was found to be similar in groups 2 and 3 (p=0.715, Table 5).

**TABLE 5:**
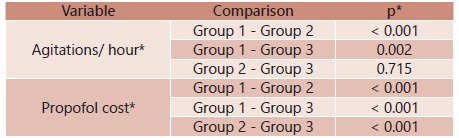
Results from multiple comparisons among induction types for procedure parameters with statistically significant difference

*.: Dunn's multiple comparison.

Regarding the cost of propofol used in this study, a statistically significant difference among all groups was found (p<0.001) presenting lower value in group 1 and higher in group 3. Among groups compared individually, this statistical difference was found to be maintained.

As for blood pressure, difference in data initially collected was found (p=0.008). However, after induction blood pressure measurements were not statistically significant across groups. In addition, no statistical differences related to initial heart rate (p=0.453), mean after induction (p=0.702), and lowest heart rate reached (p=0.788) were verified (Table 6).

**TABLE 6:**
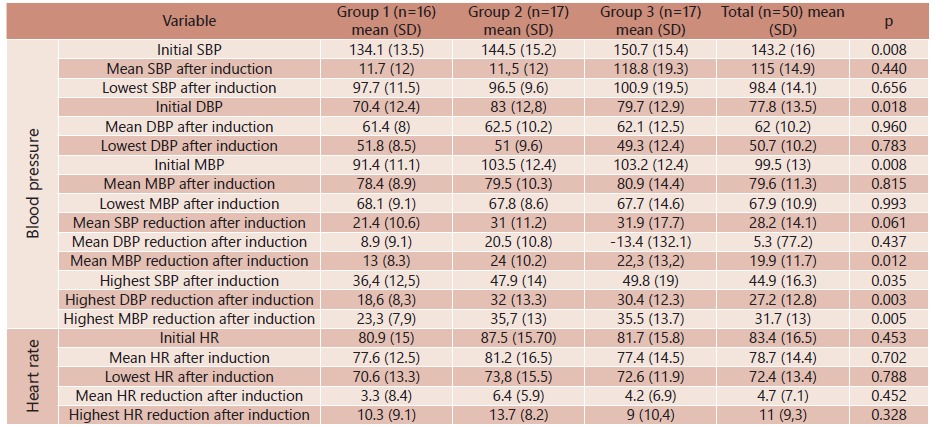
Blood pressure and heart rate data evaluated during procedure and changes of these parameters across groups and results from comparisons

HR=heart rate, SD=standard deviation; min=minimum; max=maximum; SBP=systolic blood pressure; ABP=arterial blood pressure; MBP=medical blood pressure

At the completion of examination, no patient presented with hypotension or oxygen saturation drop, so that pressure levels and heart rate were normal or suitable to blood pressure levels.

## DISCUSSION

After evaluating results individually, variability for BIS value was found to be high. BIS value varied from 17 to 72. Since that even in readings no greater than 60, when a deep sedation degree would be expected, patients still presented with agitations during examination, demonstrating that this is not an effective parameter to evaluate sedation degree of patients and only moderate correlation in the Observer's Assessment of Alertness/Sedation (OAA/S) Scale^2^.

BIS was demonstrated to have low precision to demonstrate deep sedation levels by other studies and propofol titration for determined sedation levels were not useful^4^ ^,^ ^13^.

Regarding technical difficulties described in the studies about sedation, the fact that no completely adequate catheters were available to monitor capnography stimulated the crafting of a system that circumvented this problem, a quite reliable solution to detect airway obstruction. As an intercurrent event, obstruction of the aspiration branch was frequent, temporarily interrupting the reading of capnographic waves.

Additional oxygen administration, on occasion, resulted in dilution of the sample collected, consequently affecting the reading accuracy of capnography absolute values because volume/minute variability of patients and airway anatomic changes in subjects made this value unsatisfactory with the real concentration of carbon dioxide of patients in many cases.

However, waves had good quality and enabled good readings for airway obstruction, hypopneia, and apnea, as well as the effective maneuvers to correct them, demonstrating that this is a good monitoring parameter to prevent hypoxia.

The difference in initial blood pressure is not believed to result from the infusion method or group to which patients were assigned, but actually to their individual subjective characteristics, regardless of the propofol infusion protocol adopted.

In general, blood pressure was reduced with the use of propofol corroborating the literature. In the case of a 78-year-old patient with worse clinical conditions, ASA III class, drop below 60 mmHg occurred and the use of vasopressor was required after the increase of hydration resulted to be ineffective. This result may demonstrate that a specific protocol for patients in these conditions might be tested and further applied.

Oxygen saturation drop occurred in six patients (12%) from the sample evaluated in this study and reverted in less than 5 min with maneuvers of mandible elevation or using a Guedel cannula. Before drops in oxygen saturation occurred, typical changes in airway obstruction, hypopnea or apnea were detected through capnographic waves in 16 patients (32%), so that this parameter was found to be good for monitoring hypoxia prevention in some patients, in more than one occasion, with 100% sensitivity. No difference across groups for obstruction of airways/apnea was verified (p=0.543).

Although the desired clinical effect and adverse effects associated to propofol are closely related to propofol plasma concentration, little data were found about its serum dosage during sedation^1^ ^,14^.

The similarity of values found in propofol serum dosages is corroborated by this study, as well as the clinical parameters evaluated along examinations.

In a systematic review conducted by Leslie^9^ *et al,* with 20 studies comparing manually controlled infusion with TCI, no evidence was found to justify the recommendation of one or other method in a manuscript made by the same author^8^. In this study, the similarity between both methods was demonstrated, but with preference to group 2 due to its much lower cost, taking into consideration the innovative preparation for the described solution.

In terms of cost analysis, in our study, group 1 was found to yield the lowest mean value for colonoscopies evaluated with mean expenditure of R$ 7.00 (approximately US$ 2.25); group 2, R$17.50 (approximately US$ 5.64) and group 3, R$ 112.70 (approximately US$ 36.35, p<0.001). However, differences in maximum dosages that might be administered across examination duration since its beginning were found. In group 1, the initial cost of R$ 4.90 (approximately US$ 2.25) was equivalent to 200 mg of propofol, which in the average consumption 0.12 mg/kg/min for a 70 kg patient would be enough for about 23 min of examination. In group 2, the initial propofol dose was 600 mg with average consumption of 0.13 mg/kg/min, which would be enough for about 60 min of examination. In group 3, initial propofol dose was 500 mg. Since examination duration was 34.8±18.5 min on average, a reserve of unused propofol remained in groups 2 and 3 after procedures were completed.

## CONCLUSION

Colonoscopy sedation using propofol bolus in continuous manual infusion or continuous automatic infusion was found to present similarities regarding clinical parameters evaluated and propofol plasma concentration, except for the presence of more agitation with the use of bolus. Among continuous infusion methods, the use of manual infusion yielded much lower cost. The use of an innovative adapted catheter for capnography is inexpensive and reliable for the early detection of airway obstruction during procedure.
